# Endobronchial tuberculosis: histopathological subsets and microbiological results

**DOI:** 10.1186/2049-6958-7-34

**Published:** 2012-10-22

**Authors:** Sevket Ozkaya, Salih Bilgin, Serhat Findik, Hayriye Çete Kök, Canan Yuksel, Atilla Güven Atıcı

**Affiliations:** 1 Rize University, Faculty of Medicine, Department of Pulmonary Medicine, Rize, Turkey; 2 Samsun Chest Disease and Chest Surgery Hospital, Samsun, Turkey; 3 Ondokuz Mayis University, Faculty of Medicine, Department of Pulmonary Medicine, Samsun, Turkey; 4 Samsun Pathology and Cytology Centre, Samsun, Turkey; 5 Ondokuz Mayis University, Faculty of Medicine, Department of Pulmonary Medicine, Samsun, Turkey

**Keywords:** Bronchoscopy, Endobronchial tuberculosis, Microbiology, Radiology

## Abstract

**Background:**

Endobronchial tuberculosis (EBTB) is defined as a tuberculous infection of the tracheobronchial tree with microbial and histopathological evidence, with or without parenchymal involvement. Bronchoscopic appearances of EBTB have been divided into seven subtypes: actively caseating, edematous-hyperemic, fibrostenotic, tumorous, granular, ulcerative, and nonspecific bronchitic. However, information for establishing a definite microbiological diagnosis in each of these categories is lacking.

We aimed to present bronchoscopic appearances and percentages for the EBTB subtypes and to compare bronchoscopic appearances with microbiological positivity in bronchial lavage fluid.

**Methods:**

From 2003 to 2009, 23 biopsy-proven EBTB patients were enrolled in the study. Diagnosis of EBTB was histopathologically confirmed in all patients.

**Results:**

The commonest subtype was the edematous-hyperemic type (34.7%); other subtypes in order of occurrence were: tumorous (21.7%), granular (17.3%), actively caseating (17.3%), fibrostenotic (4.3%), and nonspecific bronchitic (4.3%). Although all patients were sputum-smear-negative for acid-fast bacilli (AFB), 26% of patients were smear-positive for AFB in the bronchial lavage fluid. The bronchial lavage fluid grew *Mycobacterium tuberculosis* in 39.1% of all patients.

The bronchial lavage smear positivity for AFB in the bronchial lavage fluid was 75%, 25%, 20%, 12.5%, 0%, and 0% for the granular, actively caseating, tumorous, edematous-hyperemic, fibrostenotic, and nonspecific bronchitic subtypes of EBTB, respectively. Culture positivity for *Mycobacterium tuberculosis* in bronchial lavage fluid was 75%, 50%, 40%, 25%, 0%, and 0%, respectively.

**Conclusion:**

The commonest subtype of EBTB was the edematous-hyperemic subtype. The granular type had the highest smear positivity and culture positivity for *Mycobacterium tuberculosis* in bronchial lavage fluid. Bronchoscopy should be performed in all patients suspected to have EBTB.

## Background

Tuberculosis (TB) is a disease of poverty, affecting the most vulnerable groups of the world’s population: more than half of TB-related deaths occur in Asia, while the greatest TB burden as percentage of population is in Africa [1]. Endobronchial tuberculosis (EBTB) is defined as a tuberculous infection of the tracheobronchial tree with microbial and histopathological evidence, with or without parenchymal involvement [2]. The incidence of EBTB was reported as 5.88% in cases with pulmonary tuberculosis [3]. However, since bronchoscopy is not routinely performed in patients with tuberculosis, the true incidence of EBTB is likely underestimated. There is only one study in the literature in which bronchoscopy was performed in all patients with active pulmonary tuberculosis, and it reported an incidence of EBTB as high as 50% [4]. EBTB continues to be a clinical challenge, especially in endemic areas, since it may mimic a variety of pulmonary diseases such as bronchogenic carcinoma, pneumonia, or bronchial asthma. The clinical course of EBTB is rather variable due to the interactions among the effects of mycobacteria, host immunity, and antituberculosis drugs; any variation in these three factors may result in different clinical presentations. Chung classified endobronchial tuberculosis into seven subtypes according to bronchoscopic features: actively caseating, fibrostenotic, edematous-hyperemic, tumorous, ulcerative, granular, and nonspecific bronchitic [5]. It was also found that the classifications was valuable to predict the therapeutic outcome of EBTB [3]. However, to our knowledge, there are not enough published data comparing the subtypes of EBTB with microbiological confirmation either using acid-fast bacilli (AFB) stain and/or culture positivity for the organism in the bronchial lavage (BL) fluid. Likewise, we could not find sufficient examples of the bronchoscopic appearances of EBTB subtypes in currently published articles. Therefore, in this study we aimed to present bronchoscopic images and percentages of the EBTB subtypes and to compare bronchoscopic appearances with the microbiological positivity in BL fluid.

## Methods

The Samsun Chest Diseases and Thoracic Surgery Hospital is a reference hospital for tuberculosis in Turkey. From 2003 to 2009, 23 biopsy-proven EBTB patients were enrolled in this study. Diagnosis of EBTB was confirmed histopathologically in all patients. Patient data were gathered and evaluated retrospectively. All patients were sputum-smear-negative according to AFB staining, and fiberoptic bronchoscopy was performed in the case of suspected tuberculosis or for the differential diagnosis of tuberculosis. Premedication for the bronchoscopy was done with intramuscular atropine at 0.01 mg/kg and opioids. The pharynx and vocal cords were also anesthetized with nebulized lidocaine. A flexible bronchoscope was inserted through the nasal passage. After inspection of the airways and before the biopsies, the bronchoscope was wedged into the airway. Sterile Saline, in aliquots of 50–60 mL, was delivered via syringe into the distal airway through the suction channel of the bronchoscope. It was then quickly aspirated through a gentle suction into a collection trap. Forceps were advanced through the bronchoscope and airway to obtain biopsies from bronchial lesions (EBBx). The patients’ characteristics, including demographic data, symptoms, initial diagnoses, and radiological, bronchoscopic and microbiological features, were reviewed and evaluated retrospectively. Bronchoscopic findings were categorized according to Chung’s classifications [4]. The results are presented as mean and percentage of patients. The study was approved by the institutional review board and written consent to use their medical records was obtained from the patients.

## Results and discussion

**Figure 1:**
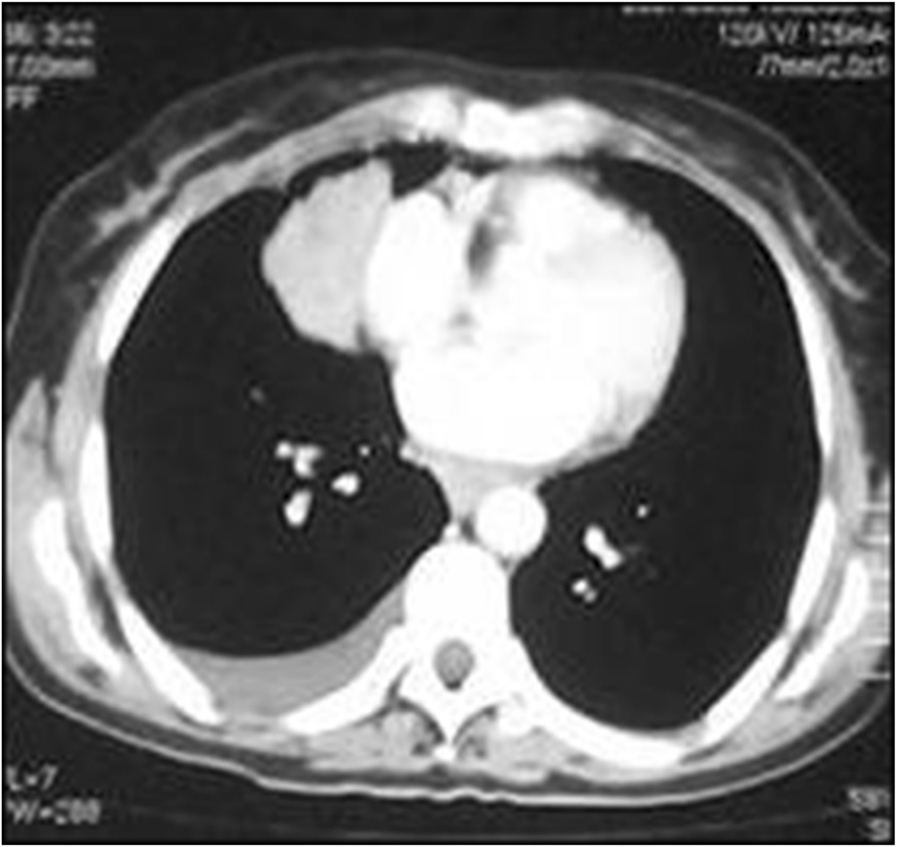
CT showing a right paracardiac intrapulmonary mass lesion and pleural effusion.

**Figure 2:**
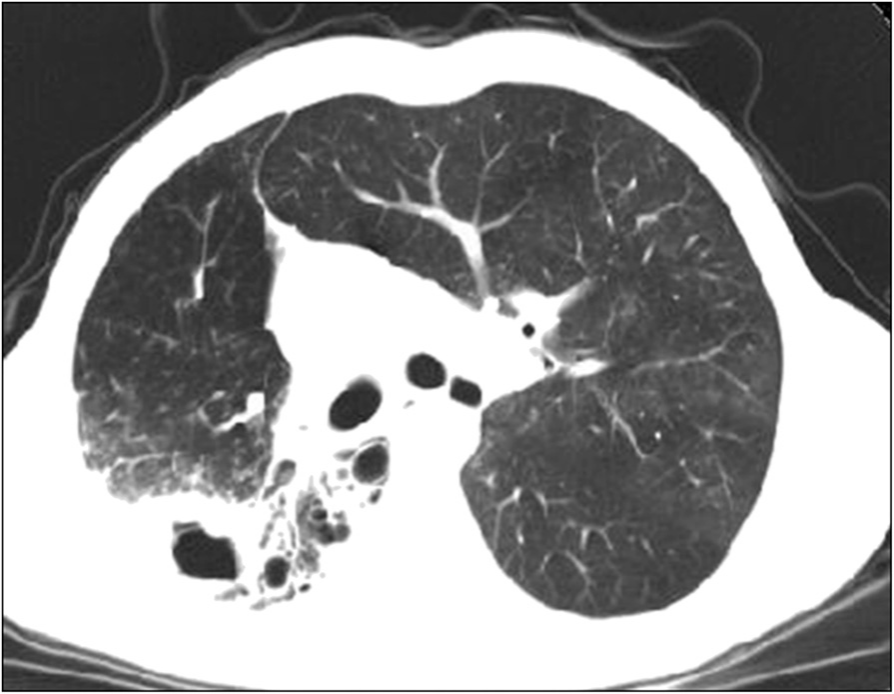
CT showing cavitation and infiltration.

During the study period a total of 1,064 patients were diagnosed with pulmonary tuberculosis at our hospital. 23 patients out of them, who were histopathologically diagnosed with EBTB via EBBx, form the study group. The incidence of EBTB was 2.2%. Demographic and clinical features are given in Table 1. The mean age of the patients was 39.6 years (range: 15–72 yrs). The female-to-male ratio was nearly 1. The commonest symptom was cough (86.9%), followed by constitutional symptoms (52.1%), dyspnea (39.1%), sputum (34.7%), fever (30.4%), and hemoptysis (13%). Initial working diagnoses were lung cancer (47.8%), community-acquired pneumonia (34.7%), and tuberculosis (17.3%). At the radiographic examination lesions were mainly located in the right lung (87%), and the commonest radiologic features were consolidation (47.8%) and mass lesion (34.7%) (Figure 1). Cavitation and pleural effusion were seen in 17.3% of patients (Figure 2).

**Table 1:** Baseline characteristics of the patients

**Characteristics**	**n (%)**
Mean age, years (range)	39.6 (15–72)
<45 years	10 (43.4)
Female/Male	12/11
Symptoms	
Cough	20 (86.9)
Sputum	8 (34.7)
Dyspnea	9 (39.1)
Hemoptysis	3 (13)
Fever	7 (30.4)
Constitutional sypmtoms*	12 (52.1)
Initial Diagnoses	
Lung Cancer	11 (47.8)
Pneumonia	8 (34.7)
Tuberculosis	4 (17.3)

* Constitutional symptoms; weight loss, weakness, anorexia.

**Figure 3:**
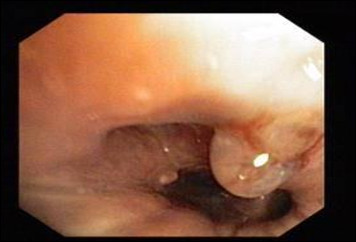
Videobronchoscopy image showing the granuler subtype of endobronchial tuberculosis.

**Figure 4:**
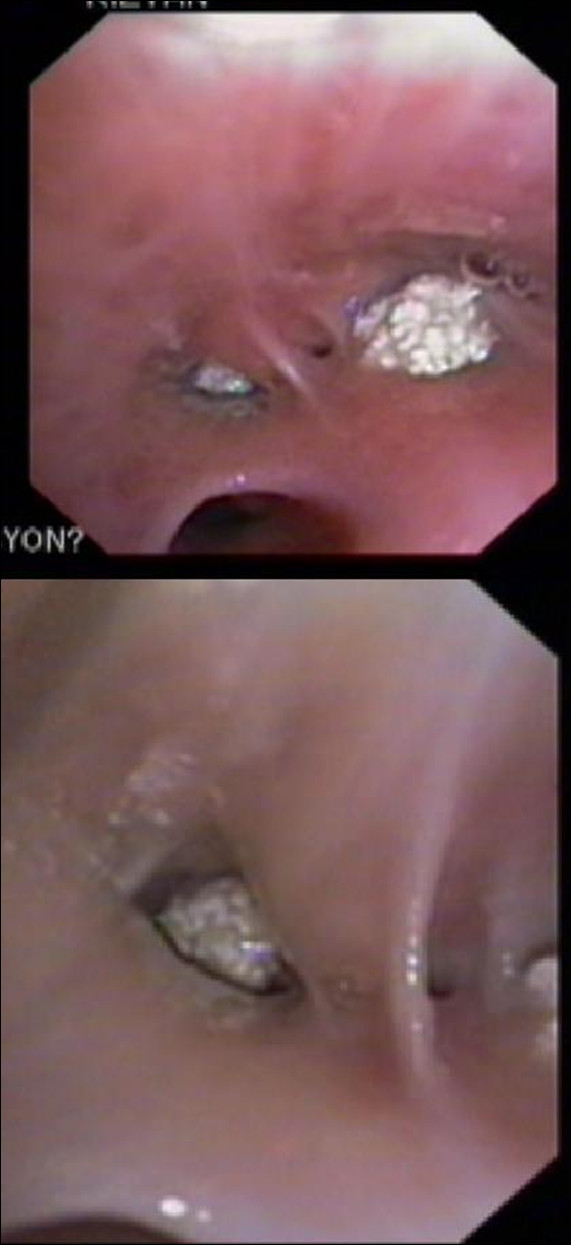
Videobronchoscopy image showing the tumorous subtype of endobronchial tuberculosis.

**Figure 5:**
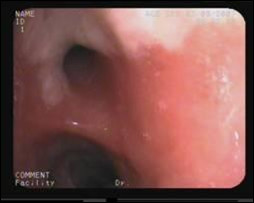
Videobronchoscopy image showing the actively caseating subtype of endobronchial tuberculosis.

**Figure 6:**
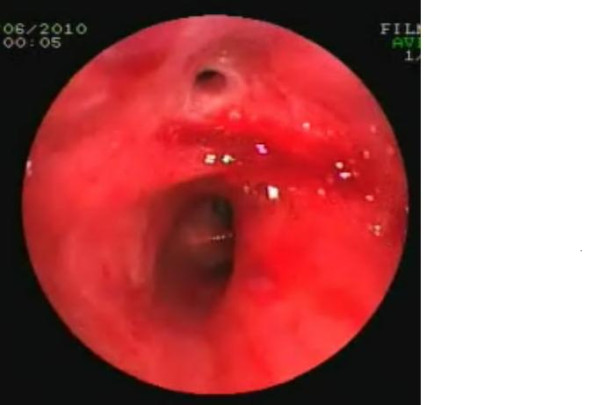
Videobronchoscopy image showing the edematous subtype of endobronchial tuberculosis.

Bronchoscopic findings were categorized according to Chung’s classifications and the subtypes were: edematous-hyperemic (34.7%), tumorous (21.7%), granular (17.3%), actively caseating (17.3%), fibrostenotic (4.3%), and nonspecific bronchitic (4.3%) (Figures 3, 4, 5, 6). No cases of the ulcerative subtype were seen in our patients (Table 2).

**Table 2:** Bronchoscopic, histopathologic and microbiologic features of patients

**Types**	**n (%)**	**Histopathology of bronchial biopsy**	**BL positivity AFB stain Culture**	
			**n (%)**	**n (%)**
Edematous,hyperemic	8 (34.7)	Caseating Granulomatous Inflammation	1 (12.5)	2 (25)
Tumorous	5 (21.7)	Caseating Granulomatous Inflammation	1 (20)	2 (40)
Granular	4 (17.3)	Caseating Granulomatous Inflammation	3 (75)	3 (75)
Caseating	4 (17.3)	Caseating Granulomatous Inflammation	1 (25)	2 (50)
Fibrostenotic	1 (4.3)	Caseating Granulomatous Inflammation	0	0
Nonspecific Bronchitic	1 (4.3)	Caseating Granulomatous Inflammation	0	0
Total	23		6 (26.1)	9 (39.1)

Table 1 legend – *AFB*, Acid-fast bacilli; *BL*, Bronchial Lavage.

Anatomically, the bronchoscopic findings were primarily located in the right upper lobe bronchus with an incidence of 47.8%, which was followed by the middle lobe bronchus (21.7%) and the right lower lobe bronchus and the left upper bronchus (13%).

Microbiologic and smear examination of BL fluid was positive for AFB in 26% of the patients. The highest smear positivity for AFB was found in granular-type cases (75%). Cultures of BL fluids for *Mycobacterium tuberculosis* were positive in 39.1% of patients, and the positivity was highest in granular-type cases (75%). The results of smears and cultures for *Mycobacterium tuberculosis* are shown in Table 2.

The histopathologic examination of bronchoscopic biopsies revealed caseating granulomatous inflammation in all patients.

The present study classifies EBTB cases according to Chung’s classification and investigates whether there are any differences in microbiologic results among these subtypes for AFB staining and tuberculosis culture. The edematous-hyperemic subtype was the commonest appearance, seen nearly in one-third of patients. While the highest smear and culture positivities for *Mycobacterium tuberculosis* in BL fluid were seen in the granular type, both tests were negative in patients with fibrostenotic and nonspecific bronchitic EBTB.

The incidence of EBTB in the present study was 2.2% in all pulmonary TB patients. This incidence may not reflect the real situation because the retrospective analysis was based on passive detection. The incidence was lower because bronchoscopy is not routinely performed in all patients with TB; the actual incidence of EBTB remains underestimated [3]. EBTB usually affects adults, including both younger and elderly patients ranging 14 to 81 years [6]. In our study the mean age was 39.6 years and 38% of patients were younger than 45 years, which was consistent with the literature. The female-to-male ratio was 12:11; EBTB was more frequent in female patients (5:1 in females and 2:1 in males). One of the possible reasons for female predominance in our region may be the fact that women usually do not expectorate sputum [5]. The commonest symptom was cough, which was consistent with previous studies.

The pathogenesis of EBTB is not fully known yet. However, possible sources of EBTB may include direct implantation of tubercle bacilli into the bronchus from an adjacent parenchymal lesion, or from an adjacent tuberculous mediastinal lymph node, hematogenous spread, erosion of an intraparenchymal tuberculous lymph node into the bronchus, or extension to the peribronchial region by lymphatic drainage [7-10].

The clinical course of EBTB is quite variable because the interactions among the effects of mycobacteria, host immunity, and antituberculosis treatment is complex; any variation in these factors may determine a change in the course of the disease [6,11]. With respect to pathology, EBTB lesions can be divided into six steps: 1) The initial lesion presents as simple erythema and edema of the bronchial mucosa with lymphocytic infiltration of the submucosa [12]. 2) Submucosal tubercle formation produces the erythema and granularity seen during bronchoscopy, and stenosis of the bronchial lumen is due to congestion and edema [13]. 3) Development of caseous necrosis with formation of tuberculous granuloma can be found at the mucosal surface [13]. 4) When the inflammation erupts through the mucosa, an ulcer is seen. 5) The ulcer of bronchial mucosa evolves into hyperplastic inflammatory polyps. 6) The endobronchial tuberculous lesion heals by fibrostenosis [7]. Chung’s classification was based on this six-step pathological course of tuberculosis, respectively represented by the 1) nonspecific bronchitic, 2) granular and edematous-hyperemic, 3) actively caseating, 4) ulcerative, 5) tumorous, and 6) fibrostenotic types [4].

The clinical course of EBTB reflects interdependent interactions among many factors because the bronchial inflammatory reactions vary for each patient. Since the clinical course of EBTB is quite variable, all patients suspected of EBTB should be subjected to sputum smear tests and culture examination for tuberculosis [6].

The yield of sputum smear for AFB is not as high as it is in parenchymal involvement, even in an optimal laboratory set up for meticulous sputum examination. In recent studies, sputum positivity in EBTB has been demonstrated to range from 16% to 53.3% [5,14,15]. In our study, sputum smears for AFB were negative in all patients. The low yield of sputum AFB smears may be due to mucus entrapment by proximal granulation tissue. Therefore, a negative sputum smear does not preclude the diagnosis of EBTB.

The second step in clinical evaluation was bronchoscopy, to examine bronchial structures and obtain specimens for diagnosis. Different bronchoscopic specimens, including biopsy samples, brushings, and washings, may be obtained [6]. A bronchoscopic biopsy is the most reliable method for diagnosing EBTB, because needle aspiration can provide only a cytological diagnosis. Bronchial biopsies may be positive in 30% to 84% of patients [14,16]. In a clinical analysis of 90 cases of EBTB in China, bronchial brushings yielded variable results, ranging from 10% to 85% [14].

To the best of our knowledge, there has been no study to date comparing the microbiologic results with the different features of EBTB. BL fluid was positive for AFB in 26% of the cases and culture positivity for TB bacilli in the fluid was positive in 39.1%. The highest positivity for AFB was found in the granular type of EBTB (75%). The highest positivity for mycobacterial culture was also found in the granular type (75%). It is thus clear that microbiologic methods were more often found to be positive for early lesions, namely in the granular and actively caseating subtypes of EBTB. We think that the granular type of EBTB may reflect the earliest granulomatous inflammatory reaction against *Mycobacterium tuberculosis*.

Since microbiologic methods were less positive in some cases, even 0% at the late fibrostenotic stage, histopathologic methods seem much more important in these cases and endobronchial biopsy is crucial for reaching a final diagnosis.

In this study, there was no difference regarding the sex of the enrolled patients, in fact the female-to-male ratio was nearly 1, differently from prior studies that showed a marked preponderance of female patients. This may be due to race or ethnicity, as all studies reporting female preponderance were from Korea and Japan [4,5,9].

There are some limitations to our study. First, because the study population was not very large, statistical methods could not be applied for comparison among subtypes. Nevertheless, general proportions allowed for comparison, 75% positivity for the granular subtype *versus* 0% for the fibrostenotic and nonspecific bronchitic subtypes. Second, since this was a retrospective study, some data might have been lost.

## Conclusions

In conclusion, this study showed that in the early granular and actively caseating subtypes of EBTB, the yield of microbiologic methods was higher than in the late tumorous and fibrostenotic subtypes. Therefore, bronchoscopic biopsy including mucosal and parenchymal examination should be added to bronchial lavage and/or brushing in order to increase diagnostic yield in patients suspected of EBTB.

## Competing interests

The authors declare that they have no competing interests.
